# Persistent increase in alpha-fetoprotein level in a patient without underlying liver disease who underwent curative resection of hepatocellular carcinoma. A case report and review of the literature

**DOI:** 10.1186/1477-7819-10-79

**Published:** 2012-05-06

**Authors:** Isidoro Di Carlo, Maurizio Mannino, Adriana Toro, Annalisa Ardiri, Antonio Galia, Giovanni Cappello, Gaetano Bertino

**Affiliations:** 1 Department of Surgical Sciences, Organ Transplantation and Advanced Technologies, University of Catania, Cannizzaro Hospital, Catania, Italy; 2 Department of Internal Medicine and Systemic Disease, Hepatology Unit, University of Catania, S. Marta Hospital, Via Messina 829, 95126, Catania, Italy; 3 Department of Pathology, Cannizzaro Hospital, Via G. Clementi 36, 95124, Catania, Italy

**Keywords:** Alpha-fetoprotein, Hepatectomy, HCC without cirrhosis, HCC

## Abstract

**Introduction:**

Alpha-fetoprotein (AFP) is an oncofetal protein produced by hepatocellular carcinoma (HCC). AFP level can also be elevated in other neoplastic or non-neoplastic conditions. An elevated AFP level has high diagnostic significance for HCC; at a level of >200 ng/mL, the probability of HCC is >90%. The aim of the present paper is to report a patient who underwent curative resection of HCC, who had a persistently elevated AFP level postoperatively but did not develop recurrence during a 2-year follow-up period. A review of the literature is also presented.

**Case report:**

An 82-year-old male was referred following a computed tomography scan showing a 160 mm diameter mass in the left lobe of the liver. This huge mass was diagnosed as HCC, arising in the absence of cirrhosis or viral hepatitis. After tumor removal, the patient’s high AFP level persisted for 2 years.

**Conclusion:**

As steatosis was the only pathological change in the remnant liver, this may have caused the persistently elevated AFP level in this patient.

## Background

Hepatocellular carcinoma (HCC) is commonly associated with hepatitis B or C virus infection, and is particularly frequent in patients with cirrhosis [1]. HCC arising in a normal liver without cirrhosis or hepatitis is rare, with few reports in the literature [2-4].

HCC causes 1 million deaths every year, making it the fifth most common malignancy worldwide, and the incidence is increasing [5-8]. The current standard of care in patients with cirrhosis includes HCC screening with six-monthly measurements of alpha-fetoprotein (AFP) level together with abdominal ultrasonography or computed tomography (CT) [9]. AFP is an oncofetal protein produced by HCC, but AFP level can also be elevated in other neoplastic or non-neoplastic conditions [1,10,11]. An elevated AFP level has high diagnostic significance for HCC. When the AFP level is >200 ng/mL, the probability of HCC is >90% [12].

If the tumor is resectable, surgery is considered to be the curative treatment choice [5,13]. Postoperative recurrence is common, and measurement of AFP level is considered to be an extremely important screening test for the early detection of recurrence [14].

The aim of the present paper is to report a patient with a normal liver who underwent curative resection of HCC, followed by a persistently elevated AFP level during two years of follow-up without recurrence. A review of the literature is also presented.

## Case presentation

**Figure 1:**
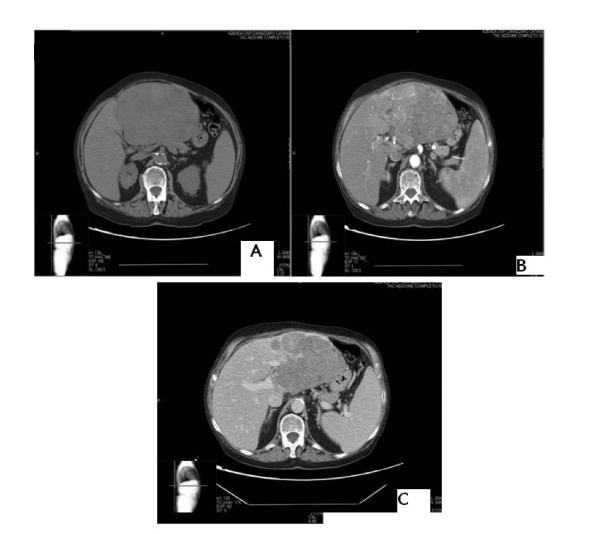
**Computed tomography scan of the tumor in liver segments 2 and 3. (A)** Pre-contrast phase; **(B)** Arterial phase; **(C)** Portal phase.

An 82-year-old male was referred to us after CT scanning showed a 160 mm diameter mass in the left lobe of the liver (Figure 1). The mass compressed the stomach and displaced the gallbladder laterally. He was diagnosed with HCC, arising in the absence of cirrhosis or viral hepatitis.

In 2007, he underwent his first CT scan, which showed hepatic steatosis and a normal-sized liver without focal lesions. In 2009, he was diagnosed with non-Hodgkin’s lymphoma which was treated with allopurinol, pyridoxine, and folinic acid.

**Figure 2:**
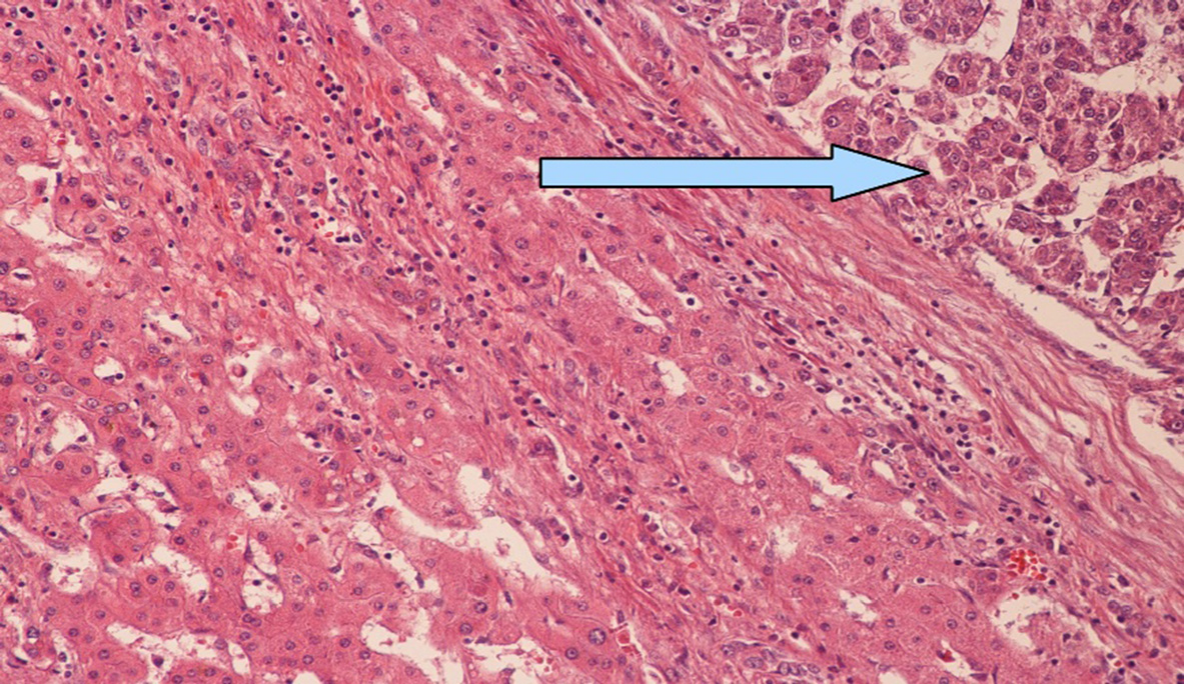
**Upper right corner: hepatocellular carcinoma (arrow).** Left: preserved laminar hepatic architecture with mild perisinusoidal lymphocytic infiltration.

**Figure 3:**
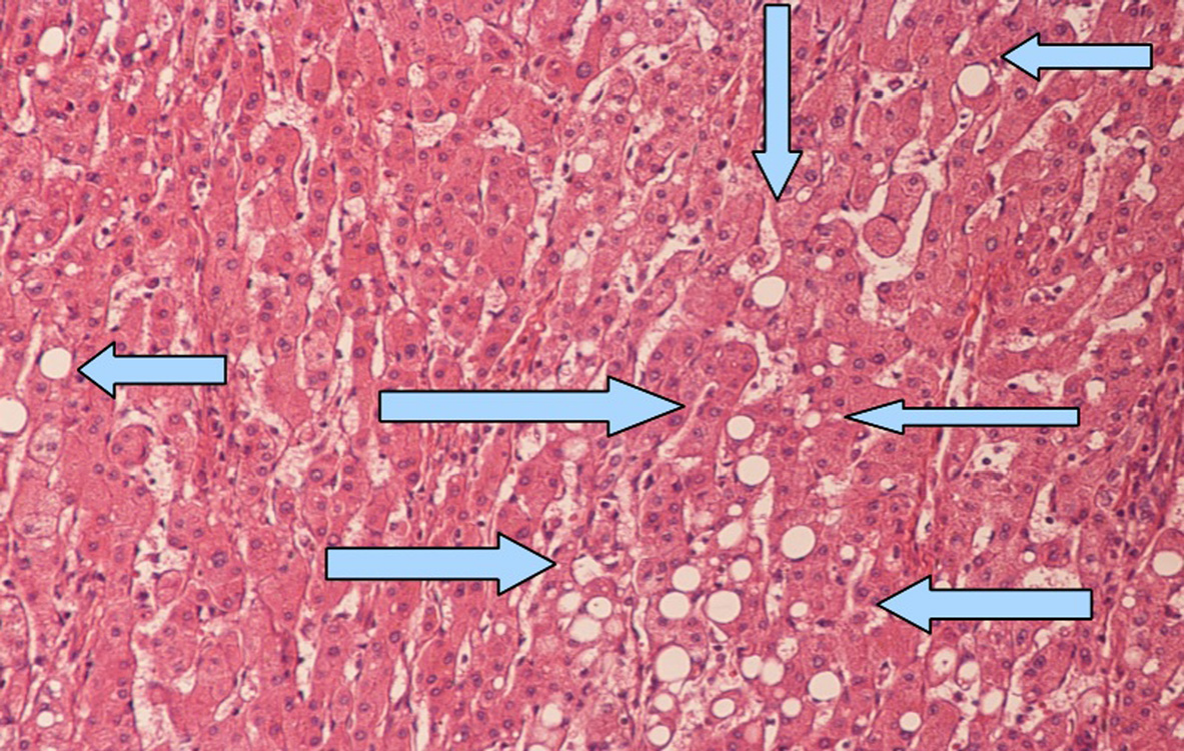
Normal hepatic tissue with diffuse steatosis (arrows).

In March 2009, he underwent the CT scan, which showed the hepatic mass and resulted in his referral to us. Magnetic resonance imaging was performed in April 2009, and showed an enlarged liver with a 168 mm mass in segments 2 and 3. He was diagnosed with HCC, and surgery was scheduled. He was in good nutritional condition, with no ascites or coagulopathy. Preoperative serum bilirubin and albumin levels were normal, and AFP level was 21 ng/mL (normal 0 to 7.22 ng/mL). He underwent open resection in April 2009. The tumor occupied all of liver segments 2 and 3, and was adherent to the stomach. The right lobe of the liver was normal in appearance. A left hepatic lobectomy was performed. Histological examination of the surgical specimen showed hepatocellular carcinoma with a solid trabecular pattern, and thick fibrotic septa which extended into the normal hepatic tissue at the resection margin (Figure 2). There was diffuse steatosis of the surrounding liver parenchyma (Figure 3).

**Figure 4:**
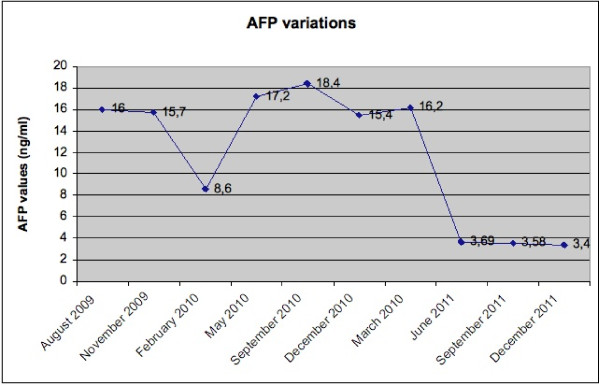
Serum AFP levels during follow-up (normal 0 to 7.22 ng/mL).

In August 2009, at the first postoperative follow-up, his serum AFP level was 16 ng/mL. Abdominal CT and positron emission tomography (PET) scans showed no evidence of tumor recurrence. Serum AFP levels were 15.7 ng/mL in November 2009 and 8.6 ng/mL in February 2010. A CT scan in March 2010 and PET scan in April 2010 did not show any evidence of recurrence. AFP levels were 17.2 ng/mL in May 2010; 18.4 ng/mL in September 2010; 15.4 ng/mL in December 2010; 16.2 ng/mL in March 2011; 3.69 ng/mL in June 2011; 3.58 ng/mL in September 2011; and 3.4 ng/ml in December 2011 (Figure 4). The patient was investigated for other conditions that might cause elevated AFP levels. Testicular, gastric, pancreatic, biliary and lung cancer, and spherocytosis and tyrosinemia, were excluded.

## Discussion

AFP is an oncofetal protein of approximately 72 kDa. AFP is produced by normal gastrointestinal cells, yolk sac cells, and fetal hepatocytes immediately after birth. Serum AFP level decreases gradually after birth, to <10 ng/mL within 300 days [15]. Some reports indicate that healthy individuals may have AFP levels of up to 20 ng/mL, and levels above this are considered to indicate the possibility of liver disease [16]_._ The normal range is given as 0 to 7.22 mg/mL in our laboratory and, in the current case, the AFP level was persistently above this range.

AFP level may be elevated in patients with chronic liver disease such as hepatitis or cirrhosis, or patients with drug or alcohol abuse, but in these cases the level is usually <100 ng/mL [11,17]. The presence of a focal hypervascular lesion >2 cm in diameter associated with a serum AFP level of >400 ng/mL is considered diagnostic for HCC [18]. The Italian and the American Association for the Study of Liver Diseases guideline considers an AFP level of ≥200 ng/mL to be diagnostic for HCC [11] .

AFP level is used for both early diagnosis and monitoring of HCC, but it is estimated that the false-negative rate when using AFP level as a single parameter may be as high as 40%, especially in patients with early HCC. AFP level may remain in the normal range in 15 to 30% of patients with HCC [19]. A high preoperative AFP level is associated with intrahepatic metastasis, early recurrence, and a poor survival rate after hepatectomy. The ratio of AFP level to tumor diameter may be a better predictor of recurrence after curative resection than serum AFP level alone [20].

AFP level can be used for postoperative monitoring, especially when it is elevated in the early postoperative period. Recurrent HCC is expected to have the same bioactivity as the primary tumor. Some patients with an elevated AFP level at initial HCC presentation do not have an elevated level with recurrence; this may be because the recurrence is actually a second primary tumor with chromosomal aberration [21].

Exacerbation of infectious hepatitis or cirrhosis may also cause a transiently elevated AFP level [22].

AFP levels vary during the different phases of the underlying disease, but do not follow a regular pattern. As AFP levels vary between patients and diseases, it is possible that two patients with the same disease, who are in the same phase of disease progression, have differences in their AFP levels; one could have an elevated AFP level, while the other could have an AFP level close to zero [11,22].

The persistently elevated AFP level after hepatectomy in the current patient cannot be explained by exacerbation of an underlying disease, because he did not have hepatitis or cirrhosis. Incomplete resection of the tumor margins might be suspected, but pathological examination showed adequate margins, and curative resection was confirmed by the absence of recurrence on CT and PET scans.

As well as being produced by the tumor, AFP may play a role in tumor growth, as preclinical evidence has shown that AFP may have a modulatory effect on hepatoma cells [20].

Many studies have reported that an elevated AFP level is an adverse prognostic factor in both early and advanced stages of HCC. An AFP level of >10,000 ng/mL is associated with a three-year survival rate of 40%, and an AFP level of 200 to 10,000 ng/mL is associated with a three-year survival rate of 70% [20]. In HCC patients with a low AFP level (20 to 100 ng/mL), the AFP level may decrease and falsely indicate a response to treatment, due to the low specificity of AFP for HCC in this range [20].

AFP levels are useful for evaluating the response to treatment and for detecting recurrence [11]. In cases of HCC arising in a liver without cirrhosis or viral hepatitis, these variations can be more evident, and normal cut-off values may be lower. Lubrano et al. [3] reported a study of 20 patients with this rare presentation of HCC, and found that only half had elevated AFP levels. As the current patient had the same type of disease as those evaluated in the study by Lubrano et al., his persistently elevated AFP level after hepatectomy, which was above the normal range but not as high as the cut-off value, was particularly suspicious for recurrent HCC and had to be carefully evaluated.

Although the literature describing HCC arising in a liver without cirrhosis or viral hepatitis is inconsistent, existing studies do agree on some aspects of this disease. The most important aspect is the difference between the natural courses of HCC arising in normal liver parenchyma and in cirrhotic liver. Liver cirrhosis and portal hypertension result in a reduced survival rate in HCC patients, especially those treated in the early stage of the disease (TNM stage I) [2,3,23]. It has also been reported that HCC in non-cirrhotic livers is more frequent in men than in women, and that the tumors are larger than those in cirrhotic livers [3,23]. In patients with HCC arising in both non-cirrhotic and cirrhotic livers, the main cause of postoperative death is cancer recurrence, which usually occurs within two years after surgery [23].

The frequency of postoperative recurrence is similar in patients with hepatitis B or C virus infection and those without, but patients without chronic hepatitis have better liver function and tend to get fewer nodules in the remnant liver, which enables easier resection of recurrences [4].

Extensive resection with a wide tumor margin is technically easier in HCC arising in a non-cirrhotic liver than a cirrhotic liver, resulting in a lower probability of recurrence and a better prognosis [23-26].

Elevated AFP level is not always associated with liver disease. Other conditions (neoplastic or non-neoplastic) can also cause a transient or persistent elevation in APF level, and should be considered in the differential diagnosis. Non-seminoma testis cancer can cause an elevated AFP level in association with elevated levels of other tumor markers such as beta-human chorionic gonadotropin and lactate dehydrogenase [27,28]. A pure seminoma may also produce AFP, but should then be managed as a non-seminoma cancer [28]. Approximately 2 to 6% of gastric cancers produce AFP, and these are generally associated with high rates of venous invasion, lymph node metastasis and liver metastasis, and an extremely poor survival rate, compared with other gastric cancers [29]. Hepatoid carcinoma is a primary extrahepatic neoplasm that has similar features to HCC in terms of immunohistochemistry, morphology, and biological behavior. Many patients with hepatoid carcinoma have an elevated serum AFP level at presentation. These tumors most commonly arise in the stomach, but a small proportion arise in the pancreas [30]. Approximately 2% of lung cancers produce AFP. AFP-producing lung cancers are usually adenocarcinomas, with a small proportion being large-cell carcinomas and squamous-cell carcinomas [31].

Pregnancy may cause an elevated AFP level, especially when the pregnancy is complicated by an abnormality such as a spinal cord defect [32].

Obesity and diabetes seem to be associated with HCC. These conditions are risk factors for cirrhosis, but their role in the non-cirrhotic liver is more controversial [26].

Hepatic steatosis can also cause a high AFP level. Steatosis can be divided into two phases; during the first phase, lipids accumulate in the hepatocytes, and in the second phase, factors such as oxidative stress, proinflammatory cytokines, mitochondrial dysfunction, and lipid peroxidation damage the hepatocytes and cause inflammation and fibrosis. In 2009, a study by Babali et al. [33] found that AFP level was associated with the grade of steatosis. In patients with steatosis, AFP production may be stimulated by cytokines, or may result from altered interactions between hepatocytes [33].

In our patient, steatosis was the only abnormality of the hepatic parenchyma detected on pathological examination, and was the only condition that could have caused the persistently elevated AFP level, as laboratory tests and imaging results eliminated all other possible causes.

Non-alcoholic steatohepatitis (NASH) can induce HCC by unknown mechanisms, in which insulin resistance seems to play an important role. Insulin resistance causes inhibition of hepatic mitochondrial fatty acid oxidation and stimulation of microsomal peroxidases, resulting in increased intracellular concentrations of fatty acids, which may cause oxidative DNA damage [34].

NASH can evolve into cirrhosis before giving rise to HCC, but the cirrhosis stage is not necessary, as there are many reported cases in the literature of HCC arising directly from NASH. NASH therefore has to be considered as a possible cause when HCC arises in a non-cirrhotic liver [34,35]. HCC arising in NASH may occur without an elevated AFP level [36].

Another rare condition that may cause an elevated AFP level in a patient with a normal liver is hereditary persistence of AFP (HPAFP), which is asymptomatic. Only 19 families with HPAFP have been described over the past 28 years, many of whom also had urological disorders (malignant or benign). HPAFP can, therefore, also be considered in patients with an unexplained high AFP level [37,38].

## Conclusions

In conclusion, we present a case in which hepatic steatosis is the only possible cause of the persistently elevated AFP level after curative resection of HCC.

## Consent

Written informed consent was obtained from the patient for publication of this case report and any accompanying images. A copy of the written consent is available for review by the Editor-in-Chief of this journal.

## Abbreviations

AFP, Alpha-fetoprotein; CT, Computed tomography; HCC, Hepatocellular carcinoma; NASH, Non-alcoholic steatohepatitis; PET, Positron emission tomography; HPAFP, Hereditary persistence of alpha-fetoprotein.

## Competing interests

All authors declare that they have no competing interests.

## Authors’ contributions

IDC was the principle investigator who prepared, organized, and edited all aspects of the manuscript, and the surgeon who performed the operation. MM supported the work of the principle investigator in writing and editing the manuscript. AT supported the work of the principle investigator in writing and editing the manuscript. AA supported the work of the principle investigator in preparing the manuscript. AG was the pathologist who performed the pathological examination of the surgical specimen. GC supported the work of the principle investigator in preparing the manuscript. GB supported the work of the principle investigator in preparing the manuscript. All authors read and approved the final manuscript.
